# Celebrate a decade of publishing: ten things in ten years

**DOI:** 10.1038/s41377-022-00716-9

**Published:** 2022-03-29

**Authors:** Jianlin Cao

**Affiliations:** Editor-in-Chief, Light: Science & Applications, State Key Laboratory of Applied Optics, CIOMP, CAS, No. 3888, Dongnanhu Road, Changchun, China

**Keywords:** Fibre optics and optical communications, Fibre lasers

It all started on 29^th^ March, 2012, the day *Light: Science & Applications* (*LSA*) was launched. The journal was established to promote research in all areas of optics and photonics, including basic, applied, and engineering research and applications, by publishing original articles, reviews, news & views, and perspectives of high quality, interest, and far-reaching consequence. Looking back on that day, our optics and photonics community may hardly imagine how thrilled every founding member of *LSA* can be, to see the inaugural papers launched. Now, ten years after its founding, *LSA* has evolved to be an essential and visible resource for our community.

**Fig. 1:**
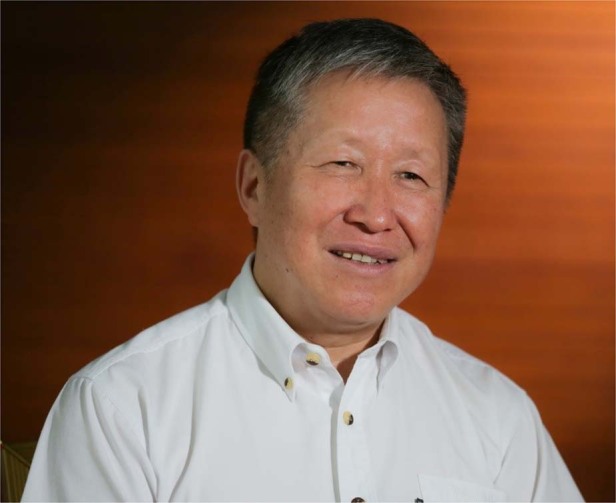
When *LSA* was founded, I was a Vice Minister of Science and Technology of the People’s Republic of China, my long experience and insight with international science collaboration made me very much desire a journal to make the exchange of scientific knowledge freely exchanged and available to all, regardless of geography. That’s my main motivation to found *LSA*.

At this moment, I am delighted to highlight ten things from the past 10 years. And quoting from the first editorial I published in 2012, because that’s exactly how I felt and what I’m feeling right now: *I put my pen to paper here, filled with emotion and enthusiasm* (Fig. 1).

**Ten things in 10 years**

1. Superb Editorial board
2. Authors, reviewers, and readers
3. Annual Light Conferences
4. Serve the optics community
5. Special Issues and columns
6. Founding partner of UNESCO’s IYL and IDL
7. Light online talks
8. Global visibility
9. New sister journals
10. Databases and sponsors

**Fig. 2:**
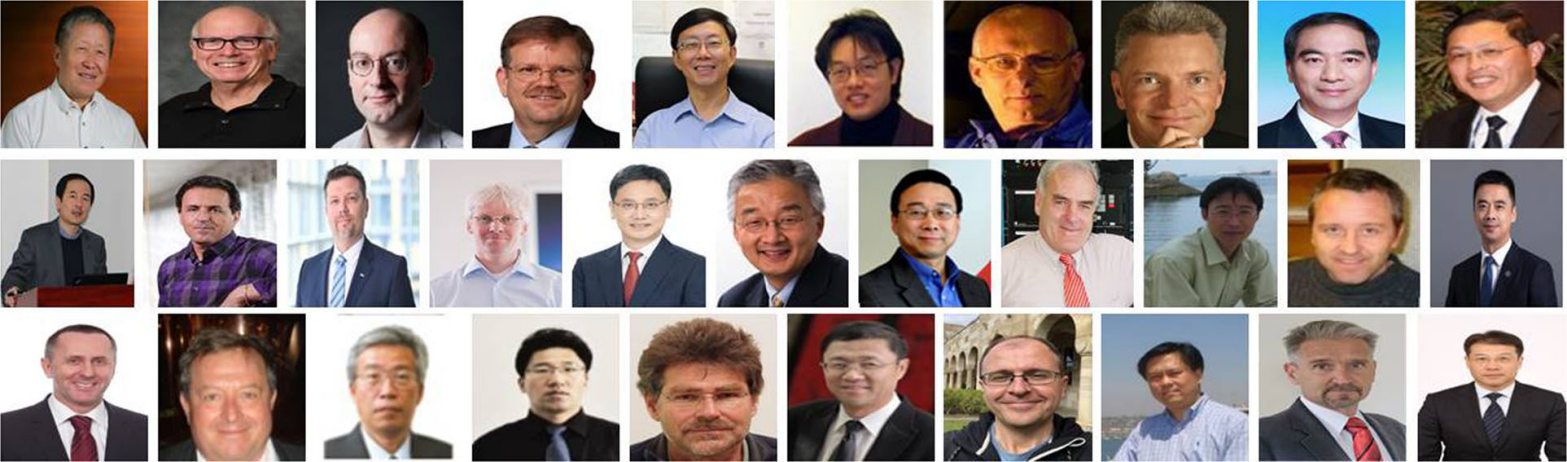
Founding Editorial Board Members of *Light: Science & Applications*.

First of all, **our Superb Editorial Board**: I’d like to express my deepest congratulations and appreciation to our editorial board members (see Fig. 2 for our founding editorial board members), and regional ad-hoc editors. Since the very beginning, *LSA* has adopted editorial board members and in-house editors as co-working mechanisms. Their dedication and efforts were instrumental in *LSA*’s success. Since 2015, *LSA* reached out and set up regional offices in leading branches of optics. Those offices were founded in Edinburg, London, Paris, Rochester, Singapore, Sydney, Beijing, Changsha, Chengdu, Foshan, Hong Kong, Nanjing, Shanghai, Tainan, Xiamen and Zhengzhou, which supplied *LSA*’s expansion with ad-hoc editors. I fully believe that the journal’s quality is dependent on the editors’ quality. With such outstanding qualifications from our editors, *LSA* achieves her new milestones.

I am deeply saddened that Prof. Mark I. Stockman from Georgia State University, Prof. John Love from Australian National University, and Prof. Tom Gregorkiewicz from the University of Amsterdam, unfortunately passed away during their editor’s term. And I’d like to express my heartfelt condolences to our beloved three friends.

**Fig. 3:**
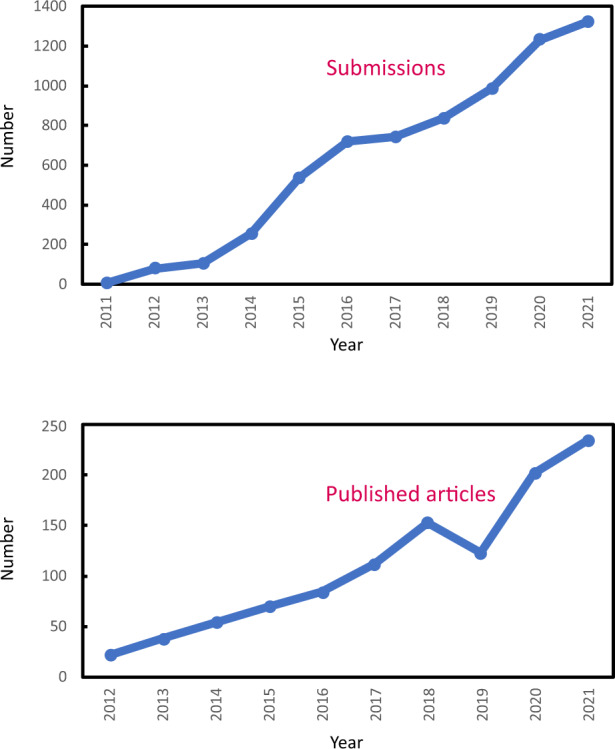
*LSA* trends on contributions and publications.

**Fig. 4:**
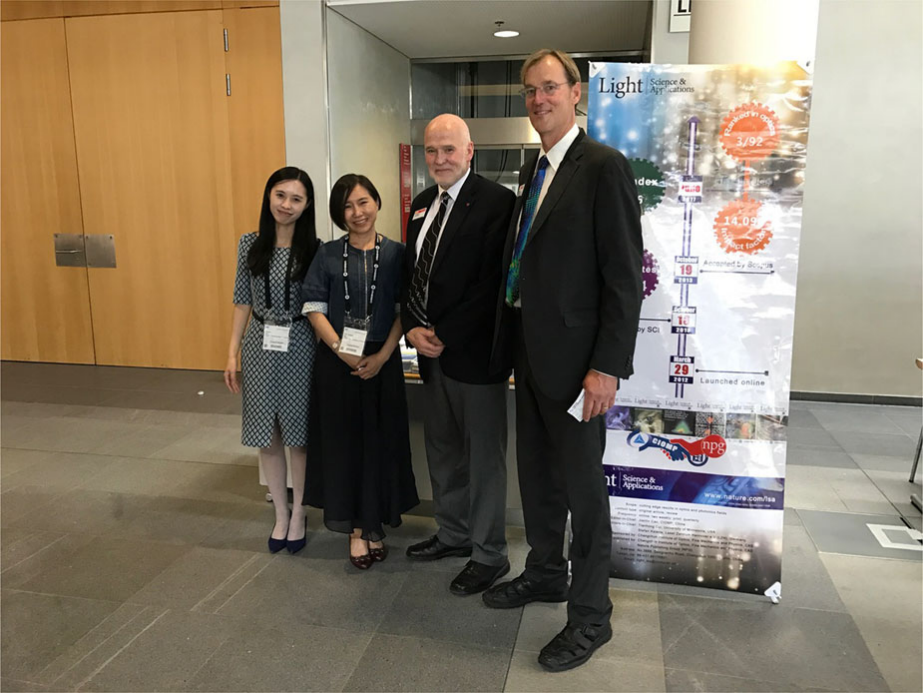
Readers at *LSA’*s booth.

**Fig. 5:**
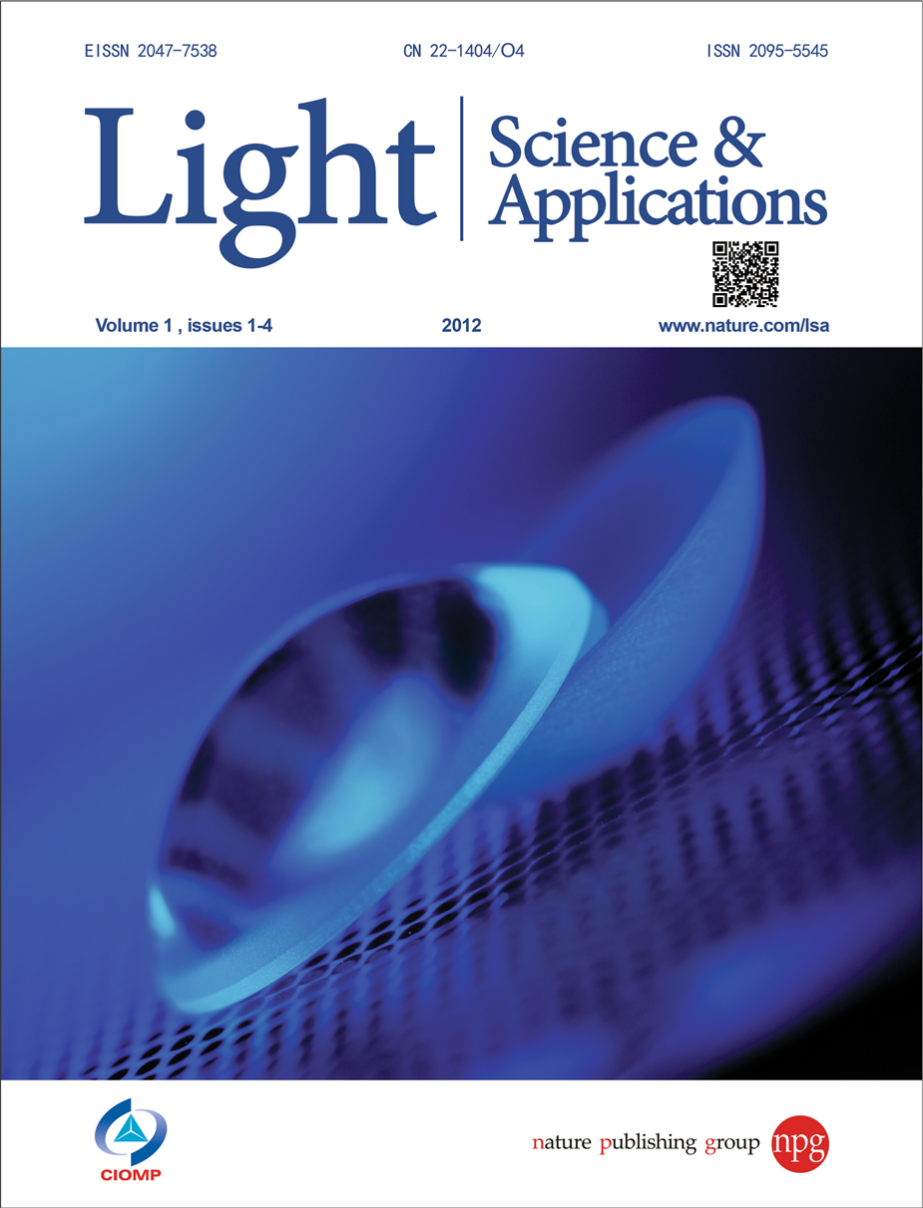
Inaugural Issue of *LSA*.

Secondly, I’m obliged to our **authors, reviewers, and readers**. During the past ten years, *LSA* has published over 1000 research and review manuscripts submitted by 1070 institutions in 58 countries/regions. It’s our worldwide authors’ high quality contributions that fundamentally nourish *LSA* and support our rapid increase in submissions (from 80 in 2012 to more than 1300 in 2021, see Fig. 3), and in publishing volume (from 22 in 2012, to 234 in 2021). Accompanied by increased submissions, inevitably, more burdens were placed on the reviewers. It’s their professional, unbiased, and timely evaluations and comments that helped improve the manuscripts to convey a better message to our broad readership. And I thank our readers for keeping a very close eye on *LSA*’s papers (see Fig. 4 for a photo of our readers with our poster, and Fig. 5 for the launching cover of *LSA*), and always sending us their first-hand feedback. For that, *LSA* might be among the few journals that issues a regular “Best Readers Award”. This award is open to anyone who makes constructive suggestions for *LSA* to improve its quality or impact, or who has evidence of promoting *LSA* in public/social/science media, conferences/webinars/workshops/forums, and published articles/books/posters/presentations.

Third, in 2011, while we were still preparing to launch *LSA*, we initiated the **Light Conferences**. This annual conference grew from friends gathering and brainstorming about science and the journal into a huge annual routine that attracted 400 attendees per year. Due to COVID-19, Light Conferences were unfortunately canceled for two years. But we hold faith that the joint endeavors of our science community will bring the day that our old friends from different countries and continents gather together in reality back to being possible.

Fourth, in addition to Light Conferences, the *LSA* initiated a number of events to benefit **the optics community**. In particular, we spared no effort to help and support young and early-career researchers. For that, we started the Light Young Scientists Award, the Light Young Scientists Forum, and a very prominent series of Rising Stars of Light. The Rising Stars of Light is a worldwide campaign for the most brilliant young scientists in optics-related topics. Since 2018, it has been hosted for four years and received worldwide recognition with more than 6,000,00 audiences, and is now listed as a featured event by UNESCO’s International Day of Light. Many of our chosen candidates have quickly established themselves and produced exciting work, with some becoming fellows of major societies and serving on the boards of top-tier journals. We’re delighted to see their contribution to the wellbeing of the optics community.

Fifth, to highlight the important topics, events, and people in optics, *LSA* launched the **Special Issues** on Information Metamaterial, Twisted Light with Orbital Angular Momentum, Topological Photonics and Beyond, Microcavity Photonics, Emerging Low-Dimensional Optoelectronic Materials and Devices, etc, and hosted Light Symposiums on different highlighting topics. The first paper in the Special Issue on Information Metamaterial—“Coding metamaterials, digital metamaterials and programmable metamaterials”—was published in 2014 and led to 1400 citations, bridging the physical and digital worlds. We opened the **column** of Light People, and Professor Donna Strickland, the 2018 Nobel Prize winner in Physics, was invited to give the first interview. The *LSA* also launched the News and Views, Perspective, and Historical Review columns to highlight important works and topics in optics.

Sixth, in 2013, the United Nations Educational, Scientific and Cultural Organization (UNESCO) proclaimed 2015 as the International Year of Light (IYL), and 2 years after that, 16^th^ May was proclaimed as the International Day of Light (IDL). With the greatest pleasure and honor, *LSA* was invited as one of the **Founding Partners of IYL and IDL**. Since then, *LSA* has been taking on duties to promote optics and photonics. At each International Day of Light (16^th^ May), *LSA* organizes highlighted events. Despite the presence of COVID-19, the 2020 Lighting the Blue Forum and the 2021 IDL conference weeks were held on-site.

Seventh*, LSA*’s been very active in the major conferences from all around the world, but COVID-19 reshaped the travel possibilities. We began to think that many of the researchers in optics might miss the talks and communication as much as we do. Therefore, we took the initiative and launched **Light Online Talks** in early 2020. Since then, more than 20 lectures and Webinars have been hosted, which attracted 1.5 million attendees.

Eighth, *LSA* has gained **high visibility all around the world**. Our editorial board members are leading scientists in optics from 18 countries. We received ~7,000 submissions from 70 countries and published papers from 58 countries. Our publications attracted millions of accesses and downloads from more than 100 countries and were widely spread through the world’s major science portals (e.g., EurekAlert!, Phys.org, etc) and social media channels (Facebook, Twitter, Instagram, and WeChat). For the past seven years, *LSA’*s impact factor has been ranked among the top three journals in the optics category, with the latest impact factor of 17.782.

Ninth, in 2021, the same team of *LSA* launched two **new sister journals** for different purposes. Launched by CIOMP and Springer Nature, *eLight* reports the emerging and multidisciplinary topics in optics, and targets groundbreaking research and only the finest works. Launched by Jihua Lab and CIOMP, *Light: Advanced Manufacturing* focuses on innovative research on light-based manufacturing. We look forward to these journals bringing huge changes to the optics publishing landscape.

Finally, I’d like to thank the **databases**—Science Citation Index (SCI), Scopus, DOAJ, Ei, PubMed, CSCD, CNKI, CSTPCD, and VINITI—for indexing *LSA* at a very early stage and helping *LSA* reach wider readers. And special appreciation to the Changchun Institute of Optics, Fine Mechanics and Physics (CIOMP), Chinese Academy of Sciences (CAS), and Springer Nature (formerly known as Nature Publishing Group), for breaking the boundaries and jointly founding *LSA*. Without their endorsement, *LSA* would not have worked from the very beginning. The final credit goes to my editorial office staff. With their extraordinary contribution, dedication and service, *LSA* departs from their safe harbor and sails into the endless oceans.

Ten years is definitely a short period in communication history, but what has been done in this period could change a new generation of researchers. Looking back at the past decade, the whole *LSA* team can proudly say that we have tried our utmost and wholeheartedly served the people in optics. And we have achieved our primary goal. We have made successful progress, some of which we could not have even dreamed of 10 years ago! I am confident that *LSA* will still be able to open our eyes wide in the future and inspire us to say, “Look, what a wonderful job *LSA* has done!”.

